# Household Costs of Diarrhea by Etiology in 7 Countries, The Global Enterics Mulitcenter Study (GEMS)

**DOI:** 10.1093/ofid/ofz150

**Published:** 2019-04-03

**Authors:** Marita Zimmermann, Karen Kotloff, Dilruba Nasrin, Anna Roose, Myron M Levine, Richard Rheingans, Tamar Farag, Damian Walker, Clint Pecenka

**Affiliations:** 1 Institute for Disease Modeling, Bellevue, Washington; 2 Department of Pediatrics, University of Maryland School of Medicine, Baltimore, Maryland; 3 Center for Vaccine Development and Global Health, University of Maryland School of Medicine, Baltimore, Maryland; 4 Department of Medicine, University of Maryland School of Medicine, Baltimore, Maryland; 5 Appalachian State University, Boone, North Carolina; 6 Institute for Health Metrics and Evaluation, Seattle, Washington; 7 The Bill and Melinda Gates Foundation, Seattle, Washington; 8 PATH, Seattle, Washington

**Keywords:** costs, diarrhea, etiology, GEMS, out-of-pocket

## Abstract

**Background:**

Although there are many overlapping features, pediatric diarrheal diseases can vary in severity, duration, clinical manifestations, and sequelae according to the causal pathogen, which in turn can impact the economic burden on patients and their families. We aimed to evaluate the household costs of diarrheal disease by pathogen in 7 countries.

**Methods:**

We analyzed data from the Global Enteric Multicenter Study (GEMS), a prospective, age-stratified, matched case–control study of moderate to severe diarrheal disease among children aged 0–59 months in 7 low-income countries; 4 in Africa (Kenya, Mali, Mozambique, The Gambia) and 3 in Asia (Bangladesh, India, Pakistan). Demographic, epidemiological, economic, and clinical data were collected, and a stool sample was obtained for microbiological analysis at enrollment. We used a multivariate generalized linear model to assess the effect of rotavirus, *Cryptosporidium*, heat-stable toxin (ST)–producing enterotoxigenic *Escherichia coli* (ETEC [ST only or LT plus ST]), *Shigella*, *Campylobacter jejuni*, norovirus GII, *Vibrio cholerae* O1, age, gender, in/outpatient, and country on total costs to the patient/family.

**Results:**

Household out-of-pocket costs were higher in Mali than any other country. Within countries, household cost differences between pathogens were minimal and not statistically significantly different.

**Conclusions:**

We found no significant differences in household costs by pathogen. Despite data limitations, understanding pathogen-specific household costs (or lack thereof) is useful, as decision-makers could consider broader illness cost information and its relevance to a particular pathogen’s economic burden and contribution to poverty when deciding which pathogens to target for interventions.

Diarrheal diseases are a leading cause of death among children in low-income countries [1]. A variety of enteric infections are responsible for most of these illnesses. The Global Enteric Multicenter Study (GEMS) identified 14 pathogens that were associated with medically attended moderate to severe diarrhea (MSD) in at least 1 age group (0–11 months, 12–23 months, and 24–59 months) among the 7 study sites in Africa (Mali, Mozambique, Kenya, and The Gambia) and Asia (Bangladesh, India, and Pakistan) [2]. Four pathogens—rotavirus, *Cryptosporidium*, *Shigella*, and *Escherichia coli* producing heat-stable toxin (ST), either alone or in combination with heat-labile toxin (designated ST-ETEC)—were associated with the majority of attributable disease. Although there is considerable overlap in the manifestations of the various etiologic agents, each bears unique virulence factors that can influence the severity, duration, nutritional consequences, and symptoms of the illness. These features, in addition to the pathogen-specific burden of disease, might influence the health care costs related to diarrheal diseases.

In many low- and middle-income countries where diarrheal disease is common, health care costs can represent a significant proportion of household expenditures. No study has yet evaluated household costs of diarrheal disease by comparing multiple pathogens and over several low- and middle-income countries. Moreover, it is not known whether these cost burdens differ by pathogen. If costs to the household differ by pathogen, it may be appropriate to target prevention efforts toward more costly pathogens in order to minimize the financial impact of the disease on families. Additionally, the feasibility of controlling or eliminating diarrheal episodes differs depending on the causal pathogen. Estimating household costs by pathogen may help determine the full economic impact of diarrheal disease control efforts at a public health level, particularly those that are pathogen-specific, such as vaccines. In this study, we aimed to evaluate the household costs of diarrheal disease by pathogen in 7 countries.

## METHODS

### Data Source

Data for this study were collected from cases enrolled in GEMS. The clinical and microbiological methods used in GEMS, and the scientific rationale, have been described in detail elsewhere [3–5]. In brief, for a 36-month period between December 2007 and March 2011, children 0–59 months of age who sought care at a sentinel hospital or health center (SHC) for acute moderate to severe diarrheal disease were enrolled as “cases.” Diarrheal disease was defined as 3 or more loose stools within the previous 24 hours and confirmed as a new episode with acute onset. MSD was defined as diarrhea with at least 1 of the following: sunken eyes, loss of skin turgor, intravenous hydration administered or prescribed, dysentery, admission or advised admission to hospital [4]. In addition, 6 of the 7 GEMS sites participated in a subsequent 12-month follow-on study referred to as GEMS-1A that used comparable methods to concomitantly investigate the etiology of less severe diarrhea (LSD) and MSD among children 0–59 months. Children with LSD were seeking care at an SHC for a new episode of acute diarrhea but did not meet the MSD definition. The GEMS analysis found that most attributable cases of MSD were due to rotavirus, *Cryptosporidium*, ST-ETEC, and *Shigella* [2]. The odds of dying during follow-up were 8.5-fold higher in patients with MSD than in controls, and pathogens associated with increased risk of case death were ST-ETEC and *E. coli* in infants aged 0–11 months, and *Cryptosporidium* in toddlers aged 12–23 months.

At study enrollment, parents or caregivers were interviewed to collect demographic, epidemiological, and clinical information; the child’s physical condition was assessed, and stool samples were collected. When the child was discharged from the SHC, his/her clinical management and outcome were documented. Although data were collected on the duration of diarrhea and clinical outcome 60 days after enrollment, they were not included in this analysis because associated cost data at that time point was not collected. Enteropathogens were identified in stool samples using standardized methods (Table 1) [5].

**Table 1.** Pathogens Identified in the GEMS and GEMS-1A Population

Pathogen	Types (Includes Species, Species, Serotypes, Pathotypes, and Genotypes)
*Salmonella*	Typhi, paratyphi, nontyphoidal
*Shigella*	*flexneri* (15 subserotypes) *sonnei* (1 serotype)*, dysenteriae* (14 subserotypes)*, boydii* (19 subserotypes)
*Campylobacter*	*jejuni, coli*
*Aeromonas*	—
*Vibrio cholera*	O1 Inaba, O1 Ogawa, O139
*Escherichia coli*	ETEC: either *eltB* for LT, *estA* for ST, or both
	Enteroaggregative: *aatA*, *aaiC*, or both
	EPEC: typical EPEC (*bfpA* with or without *eae*) or atypical EPEC (*eae* without either *bfpA*, *stx*1, or *stx*2)
	EHEC: *eae* with *stx*1, *stx*2, or both, and without *bfpA*
Rotavirus	—
Adenovirus	Serotypes 40 and 41
Norovirus	Genotypes I and II
Sapovirus	—
Astrovirus	—
*Cryptosporidium*	—
*Giardia*	—
*Entamoeba histolytica*	—
*Clostridium difficile* toxin [1]	Toxin, no toxin, no pathogen but toxin, GDH-positive toxin-negative, GDH-negative toxin-positive
*Ascaris lumbricoides* [1]	—
*Strongyloides stercoralis* [1]	—
Hookworm [1]	—
*Bacteroides fragilis* [1]	Toxin-positive

Abbreviations: EAEC, enteroaggregative *Escherichia coli*; EHEC, Enterohaemorrhagic *Escherichia coli*; EPEC, enteropathogenic *Escherichia coli*; ETEC, enterotoxigenic *Escherichia coli*; GDH, glutamate dehydrogenase; GEMS, Global Enteric Multicenter Study; LT, heat-labile toxin; ST, heat-stable toxin.

During the enrollment interview, the caregiver was asked about health care utilization for the diarrheal episode before the SHC visit. The caregiver estimated his/her total out-of-pocket expenses for the child’s medical care and transportation to health care providers, including pharmacies, traditional healers, unlicensed practitioners/village doctors/bush doctors, licensed practitioners/private doctors, shops or markets providing remedies and medicines, and other hospitals or health care centers. When the child was discharged from the SHC, the caregiver was asked about out-of-pocket expenses for care that the child received at the SHC, including costs for transportation to the hospital or clinic, consultations, drugs, diagnostics (specific diagnostic undefined, therefore no necessary lab tests for specific pathogens), food, or other costs. Caregivers were also asked about the origin of the money used to cover these expenses and earnings lost due to seeking or providing care during the child’s illness.

### Study Design

We used a retrospective cohort design for this analysis, selecting only cases from the GEMS case–control study and performing a cohort analysis on the selection. We included any patient in our analysis with nonmissing cost data who was classified as an MSD case in GEMS and GEMS-1A and an LSD case in GEMS-1A.

GEMS measured the presence of multiple pathogens (Table 1). A priori, we chose 7 pathogens of interest due to their significance in causing disease and interest in their related costs. The pathogens included were rotavirus, *Cryptosporidium*, ST-producing ETEC (ST only or LTST), *Shigella*, *Campylobacter jejuni*, norovirus GII, and *Vibrio cholerae* O1. We selected these pathogens due to their potential for treatment or prevention, hypothesized influence on costs, and/or prevalence. Pathogens common in other settings, for example, Salmonella or amebiasis, represented a small or nonexistent attributable fraction of diarrheal disease in this population and were therefore not included in this analysis.

The outcome of interest was total costs to the household. To standardize cost data, we inflated all costs in local currency to the year 2012, the final year of enrollment. We then converted local currency in the year 2012 to 2012 US dollars (USD) using consumer price index data. To calculate total household costs, we summed all out-of-pocket costs due to the episode of diarrhea, both before and during the visit, including medical costs, transportation, and lost wages. We also included time costs for any reported work time missed for transportation or providing care. To include time costs, we summed all lost wage days and multiplied by the gross domestic product per capita in 2012 USD, divided by 240 working days per year. Finally, we included the costs of prescriptions given for treatment at home. Country-specific prescription costs were not available, so these costs were based on international estimates [6]. The treatments, doses, and prices applied are shown in Appendix Table 1. We assumed 80% of those who received a prescription would purchase the treatment. We removed any outliers with >$1000 in total out-of-pocket costs (<0.1% of patients, The Gambia n = 3, Mali n = 1, Mozambique n = 4, India n = 1, Pakistan n = 2).

### Statistical Analysis

We examined baseline characteristics for the study population, including age, gender, loose stool characteristics, duration of disease before visit, and other symptoms. We then tabulated the presence of each of the 7 pathogens of interest in the study population. We also tabulated costs for inpatients and outpatients, separated by pathogen and country, and tabulated component costs for drugs, consultation fees, diagnostics, food, transportation, lost earnings, cost of the diarrheal episode before the study, and other costs, by pathogen and country. Costs before the study included any drugs, doctor visits, hospitalization, and transportation related to the diarrheal episode but incurred before study enrollment. Additionally, we compared the means and standard deviations of work time lost and of length of stay for patients with each pathogen in each country.

To adjust for the presence of multiple pathogens, we used a 2-part model with a logit function for any costs, followed by a generalized linear model with gamma family and log link. If too few patients had 0 costs to calculate margins for the probability of any costs, we assumed the probability of having costs was 1 and used a generalized linear model alone. The model was adjusted for the 7 pathogens, age group, gender, and country. We ran the 2-part model overall, adjusting for country, and additionally ran the model for each country independently.

Inpatient vs outpatient status and MSD vs LSD were mediating variables, so we did not adjust for them in the models described above. We did perform separate 2-part models to assess the effects of these 2 variables on cost by pathogen. We ran a 2-part model for MSD inpatients, MSD outpatients, and LSD patients (all LSD patients were outpatients). These models were adjusted for the 7 pathogens, age group, gender, and country. Dysentery (bloody diarrhea) and the days of diarrhea from onset before the visit were also considered mediators and were not included in the models. As a sensitivity analysis, we completed the 2-part model with measures of severity (MSD/LSD, inpatient/outpatient, dysentery, and days of diarrhea from onset to enrollment) included as confounders.

We calculated 95% confidence intervals based on the product of 1.95 times the SD for each part of the 2-part model. We also analyzed the model using time costs as the outcome to assess the impact of pathogens on costs of time only.

## RESULTS

### Patient Characteristics

During the 36-month GEMS and 12-month GEMS-1A study periods, a total of 15 303 cases (9439 cases in GEMS and 5864 cases in GEMS-1A) were enrolled, of whom 12 071 MSD cases and 3157 LSD cases had cost data available and were included in this analysis. The characteristics of the study population are shown in Table 2 for LSD and MSD cases. Patients were selectively enrolled to be distributed among 3 age strata: infants 0–11 months (38.9% for LSD, 42.0% for MSD), toddlers 12–23 months (34.7% for LSD, 33.8% for MSD), and children 24–59 months (26.4% for LSD, 24.1% for MSD); 47.0% for LSD and 43.6% for MSD were female. Most patients had loose stools at a frequency of 3–5 stools per day; 78.9% of MSD cases had sunken eyes, 34.9% of LSD and 59.0% MSD cases had fever, 20.1% of LSD and 37.7% of MSD cases had vomiting, and 23.8% of MSD cases had blood in stools.

**Table 2.** Characteristics of the Study Population at the Time of Enrollment

	Less Severe Diarrhea		Moderate to Severe Diarrhea	
	Total No. (n = 3157)	%	Total No. (n = 12 071)	%
Age group				
Infant	1227	38.9	5074	42.0
Toddler	1095	34.7	4084	33.8
Child	835	26.4	2913	24.1
Female	1484	47.0	5265	43.6
Maximum loose stools				
≤6/d	2453	80.6	7411	61.4
7–10/d	409	13.0	3428	28.4
>10/d	205	6.5	1232	10.2
Type of loose stool				
Simple watery	2430	77.0	7042	58.3
Rice water	186	5.9	607	5.0
Sticky/mucoid	541	17.1	3247	26.9
Bloody	0	0	1175	9.7
Symptoms before enrollment visit, by parental report				
Fever	1101	34.9	7109	59.0
Vomiting	633	20.1	4550	37.7
Blood in stool	0	0	2868	23.8
Lethargy before visit	384	12.2	4262	35.3
Loss of consciousness	6	0.2	231	1.9
Symptoms clinician observed at time of enrollment				
Sunken eyes	5	0.2	9530	78.9
Skin pinch slow return (≤2 seconds)	0	0	2167	18.0
Skin pinch very slow return (>2 seconds)	0	0	136	1.1
Restless/irritable metal status	639	20.2	3598	29.8
Lethargic/unconscious	17	0.5	888	7.4
No. of days of diarrhea before and including enrollment day				
1	385	12.2	1387	11.5
2	1431	45.3	3994	33.1
3	782	24.8	3621	30.0
4	291	9.2	1627	13.5
5	164	5.2	842	7.0
6	77	2.4	473	3.9
7	24	0.8	123	1.0
Inpatient	0	0	2172	18.0
Treatment				
IV	2	0.1	1549	12.8
Antibiotics	1835	58.1	9576	79.3
Oral rehydration solution	3032	96.0	11428	94.7

Abbreviation: IV, intravenous.

### Univariate Results

Detection of the 7 pathogens of interest is shown in Table 3 by country and overall. Rotavirus (14.6%), ST-producing ETEC (9.8%) and *Cryptosporidium* (8.7%) were the most prevalent pathogens for LSD, and rotavirus (17.7%), *Shigella* (11.4%), and *Cryptosporidium* (11.2%) were the most prevalent pathogens for MSD.

**Table 3.** Prevalence of Cases From Whom Pathogens of Interest Were Isolated for Less Severe Diarrhea and Moderate to Severe Diarrhea in Each Country

Pathogen	The Gambia	Mali	Mozambique	Kenya	India	Bangladesh	Pakistan	Total
Less severe diarrhea, total No. of patients	553	691	429	0	572	411	501	3157
Percentage with pathogen								
Rotavirus	11.8	9.7	22.8	0.0	6.3	32.1	12.4	14.6
*Cryptosporidium*	11.6	8.3	14.5	0.0	5.6	3.9	9.0	8.7
ST-producing ETEC	12.5	5.4	14.5	0.0	4.0	8.0	16.8	9.8
*Shigella*	3.4	0.3	3.5	0.0	1.4	6.8	6.8	3.4
*Campylobacter jejuni*	0.9	0.4	1.4	0.0	6.5	3.4	21.8	5.5
Norovirus GII	8.3	2.9	2.1	0.0	5.2	13.4	8.4	6.4
*Vibrio cholerae* O1	0.0	0.0	0.0	0.0	0.5	0.5	1.4	0.4
Moderate to severe diarrhea, total No. of patients	1380	2713	788	1778	2102	1712	1597	12 070
Percentage with pathogen								
Rotavirus	18.3	12.9	31.0	14.2	22.5	15.7	18.1	17.7
*Cryptosporidium*	12.2	10.3	16.6	11.0	13.7	6.5	11.3	11.2
ST-producing ETEC	10.9	5.1	7.2	9.9	5.7	2.9	10.2	7.1
*Shigella*	10.4	1.8	5.3	7.3	5.1	42.3	11.2	11.4
*Campylobacter jejuni*	2.5	1.8	2.0	9.6	12.4	13.1	24.6	9.5
Norovirus GII	10.4	2.1	2.5	5.0	6.5	3.5	8.1	5.2
*Vibrio cholerae* O1	0.0	0.0	1.5	0.4	2.8	1.1	6.8	1.7

Abbreviations: ETEC, enterotoxigenic *Escherichia coli*; ST, heat-stable toxin.

The unadjusted average out-of-pocket costs per patient for each of the pathogens of interest are shown in Appendix Table 2. Household costs tended to be higher in Mali, Kenya, and Bangladesh. For most countries and pathogens, standard deviations were larger than either the mean or median costs. Mean household costs were higher than median costs, indicating that the distribution of the data was skewed toward higher costs. Overall, 14.4% of patients were inpatients, though the frequency of admission ranged from 0.6% in Pakistan to 41.9% in Mozambique (Appendix Table 2). Inpatient costs were higher than outpatient costs. Inpatient costs were particularly high in Mali.

Lost earnings costs were the highest component cost and were highest in Kenya and lowest in Mali and Pakistan (Appendix Table 2). The next highest component costs were costs for treatment at home, drug costs at the center of enrollment, and costs before visiting the center of enrollment. Consultation costs, diagnostic costs, food costs, transportation costs, and other costs were generally under $0.60 on average for all countries and pathogens, with few exceptions. The most commonly reported other costs were registration (n = 347), cost of care (n = 143), and treatment book (n = 84), indicating that cost categorizations were imperfect and consultation costs were likely underestimated.

Average length of stay ranged from 2.0 (India) to 6.2 (Mali) days, and lost work time ranged from 0.1 (Mali) to 2.4 (Kenya) days (Appendix Table 2). We observed differences in length of stay in a health center and lost work time by country, though not by pathogen (data on file).

### Multivariate Results

**Figure 1.**
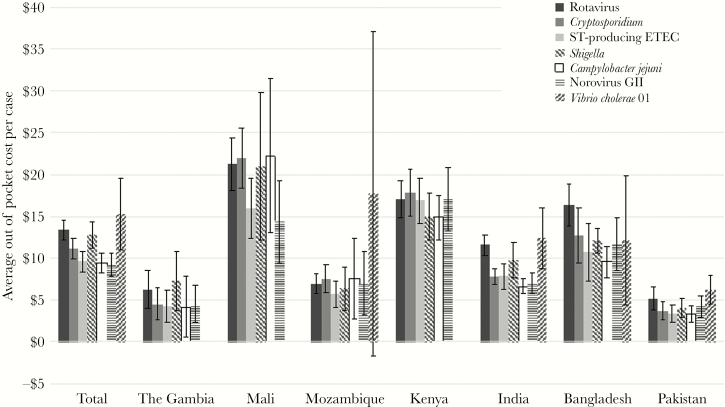
Expected out-of-pocket costs overall and by country after adjustment for other pathogens, age group, and gender. Error bars represent 95% confidence intervals. Abbreviations: ETEC, enterotoxigenic *Escherichia coli*; ST, heat-stable toxin.

Expected adjusted total out-of-pocket costs for a patient with each pathogen in each country are shown in Figure 1 after adjustment for presence of 6 other pathogens, country, age group, and gender. Total expected household out-of-pocket costs ranged from $9.15 for norovirus GII to $15.32 for *Vibrio cholerae* O1. Household costs were highest in Kenya and Mali, ranging from $14.88 for *Campylobacter jejuni* to $17.87 for *Cryptosporidium* in Kenya and from $14.41 for norovirus GII to $22.25 for *Campylobacter jejuni* in Mali. Expected household out-of-pocket costs were lowest in The Gambia and Pakistan, ranging from $4.21 for *Campylobacter jejuni* to $6.29 for rotavirus in The Gambia and from $3.32 for *Campylobacter jejuni* to $6.31 for *Vibrio cholerae* OI in Pakistan. Results of regression models can be found in Appendix Tables 3 and 4. Statistically significant regression coefficients for pathogens in these results imply that household costs are significantly different for those with the pathogen compared with those without, but they do not compare between pathogens.

Within countries, household cost differences between pathogens were, for the most part, minimal and were within the 95% confidence intervals of each other, indicating nonsignificant differences between countries (Figure 1). Using time costs (ie, lost productivity) as the outcome variable produced similar results that did not significantly differ by pathogen. Error bars in Figure 1 represent 95% confidence intervals from multiple regression analysis, calculated as 1.95 times the standard deviation. Large standard deviations can lead to larger error bars crossing the threshold of $0 costs.

**Figure 2.**
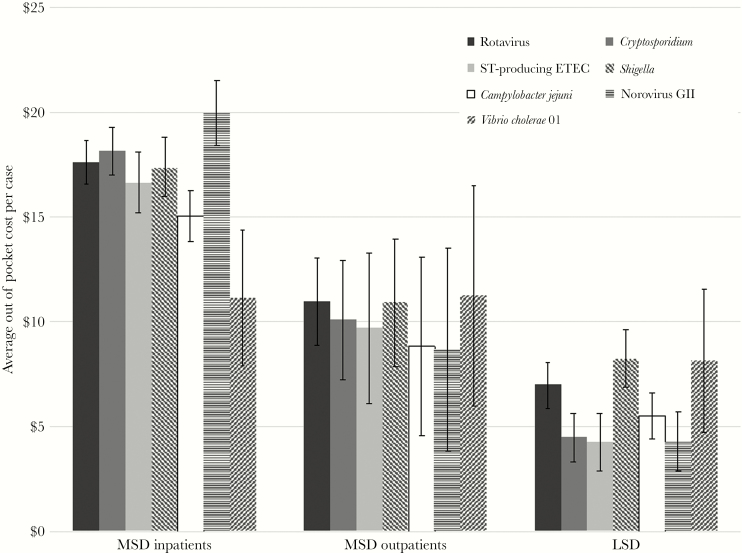
Expected out-of-pocket costs for moderate to severe diarrhea (MSD) inpatients, MSD outpatients, and less severe diarrhea patients after adjustment for other pathogens, age group, and gender. Error bars represent 95% confidence intervals.

Expected adjusted total out-of-pocket costs for inpatient and outpatient MSD and LSD patients with each pathogen are shown in Figure 2. MSD inpatients had higher costs than MSD outpatients, and MSD patients had higher costs than LSD patients. Within each of these groups, the expected cost for each pathogen was within the 95% confidence intervals of at least 1 other pathogen and often all other pathogens (Figure 2). Statistically significant regression coefficients (Appendix Tables 3 and 4) for pathogens imply that costs are significantly different for those with the pathogen compared with those without, but they do not imply a statistically significant difference between pathogens. In the sensitivity analysis adjusting for measures of severity as confounders, expected costs for each pathogen were similar to those presented above (data on file).

## Discussion

Among this population of children in 7 low- and middle-income countries, we found that unadjusted mean household costs of diarrheal disease by pathogen ranged from $8.19 for norovirus GII to $12.83 for rotavirus. Upon adjustment for presence of 6 other pathogens, country, age group, and gender, we found that household costs were higher in Kenya and Mali and lower in The Gambia and Pakistan. Within countries, expected household costs for each pathogen were within 95% confidence intervals of each other, indicating no significant difference in household costs by pathogen. We also found that household costs were highest for MSD inpatients followed by MSD outpatients, and costs were lowest for LSD patients. Although we did not compare costs for patients without the pathogens of interest, we would assume that those without pathogens causing severe disease would have lower costs. Some of the differences in household costs by country may be due to differences in overall costs and the distribution of costs between the health system and the household, but we are unable to conclusively identify the reason for these differences in this analysis. Overall, mean out-of-pocket costs are driven most by lost earnings. However, in Mali, drug costs and hospital costs are bigger drivers. In India and Pakistan, doctor and pharmacy costs before the study are also important drivers.

All cases enrolled in GEMS belonged to a censused population at each study site. Health care costs for diarrheal disease were also estimated in these censused populations in preparation for the GEMS case–control study using a Healthcare Utilization and Attitudes Survey of a randomly selected sample of the censused population stratified according to the same age categories used for cases [7, 8]. When a caregiver reported that his or her child had a diarrheal illness in the previous 2 weeks, they were asked to estimate the health care expenses that had been incurred for that illness. This retrospective report estimated the mean costs per any episode of diarrhea to be $2.63 in The Gambia, $6.24 in Kenya, $4.11 in Mali, $1.82 in Bangladesh, $3.33 in India, and $6.47 in Pakistan, with direct medical costs accounting for less than half of these costs [7, 8]. Though these results were not adjusted for confounders, were not stratified by pathogen, and generally covered a longer period of time since diarrhea onset, they are lower but of the same order of magnitude as our results. Like our study, prior analyses found higher costs in Kenya and lower costs in The Gambia. However, they also found higher costs in Pakistan and lower costs in Mali, which is contrary to our findings. These differences may be due to the inclusion of all diarrheal cases (MSD, LSD, and those not resulting in consultation at a health care center), the lack of adjustment for confounders, the small sample size, or the encompassing of more than just household costs. Prior studies also collected cost data for cases incurred in the prior 2 weeks, whereas this analysis collected cost data at enrollment and facility discharge. Our study added the examination of costs for each pathogen, which can be used in combination with the attributable fraction reported in GEMS to analyze effects of diarrheal disease on families more broadly [2]. Additional studies have examined various aspects of the costs of diarrheal disease, as in Colombia [9], Libya [10], Vietnam [11, 12], India [13–16], Rwanda [17], Kenya [18], China [19], Tunisia [20], and Kazakhstan [21]. Costs studies have also focused on the impacts of differing aspects of care [22, 23] or impacts on family budgets [24]. Finally, many costs studies have evaluated the costs of diarrheal disease in the context of cost-effectiveness of a rotavirus vaccine [25–28] or other interventions [29]. Cost of illness has been shown to vary widely by country, particularly the portion of costs incurred by the family.

There are several limitations that may have prevented this analysis from differentiating costs by pathogen. First, our analysis was limited by the lack of cost data from the facility, meaning that we could examine only costs to the household, not the total cost of a diarrheal episode. There could be differences in the costs to the facility for providing different types or levels of care, which we were not able to examine in this analysis. Second, we are limited by costs incurred only before and during the visit to the health care facility. Additional costs related to diarrheal disease may have been incurred after the visit. Therefore, our estimates should not be used to understand the full economic burden of diarrheal disease and may differentially underestimate the burden of pathogens associated with longer illnesses. Third, we lacked country-specific prescription costs; therefore, we incorporated intercountry differences due to prescribing rates but not unit costs. Fourth, although we utilized sophisticated statistical techniques to differentiate costs by pathogen, numerous pathogens were present in stool samples, and we were unable to conclusively identify any single causal pathogen for a specific diarrhea episode, which may limit the variation in costs by pathogen. We also note that our data were collected approximately 10 years ago, meaning that the values in this analysis may no longer be accurate measures of household costs. This does not affect our ability to examine costs by pathogen, which is the main objective of this paper, but caution should be exercised if one were to consider these values relative to more recently collected data. Finally, we selected pathogens a priori that were likely to cause more severe disease. Although we did include both MSD and LSD cases, we likely did not see as much variation in disease severity as we would have if all pathogens had been included. Although this may have limited our ability to detect cost differences between other pathogens, our goal was to assess meaningful differences between pathogens of interest, not to examine all potential pathogens.

Despite these limitations, an understanding of pathogen-specific differences in household costs (or lack thereof) is useful, as decision-makers could consider broader illness cost information and its relevance to a particular pathogen’s economic burden and contribution to poverty when deciding which pathogens to target with vaccines, drugs, or other pathogen-specific interventions. This study suggests that hospitalization is a key driver of diarrheal costs, though we did not identify significant differences by pathogen, because management of diarrhea tends to consist of rehydration and antibiotics. Our finding of considerable household costs for diverse diarrheal pathogens also highlights the importance of integrated intervention approaches that help address many diarrheal pathogens, such as nutrition and improvements in water, sanitation, and hygiene alongside vaccines.

## Appendix

**Table:** **Appendix Table 1.** Treatments, doses, and prices used to calculate costs of prescriptions given for treatment at home

Treatment	Assumed Package Size	Cost^a^
Oral rehydration salts	10 packets	$0.85
Cotrimoxazole	100-mL bottle	$0.48
Gentamycin	2 mL 10 mg/mL	$0.176
Chloramphenicol/thiamphenicol	100-mL bottle	$0.78
Erythromycin	100-mL bottle	$0.82
Azithromycin	15-mL bottle	$0.90
Penicillin	100-mL bottle	$1.00
Amoxicillin	100-mL bottle 125 mg/5 mL	$0.46
Ampicillin	100-mL bottle	$0.50
Nalidixic acid	500 mg	$0.0435
Ciprofloxacin/norfloxacin/other fluoroquinolone	14 tablets 250 mg	$0.30
Zinc	60-mL bottle 20 mg/5mL	$0.168
Antimalarial	100-mL bottle (chloroquine)	$1.15
Other medicine	Assumed	$0.90

^a^Source: Median price from Management Sciences for Health International Medical Products Price Guide; http://www.msh.org/resources/international-medical-products-price-guide (accessed August 8, 2018) [6].

## Appendix

**Table:** Appendix Table 2. Unadjusted Average Total, Inpatient, Outpatient, and Component Out-of-Pocket Costs for Patients With Each Pathogen, Overall and by Country

	Overall	Gambia	Mali	Mozambique	Kenya	India	Bangladesh	Pakistan
Total costs,^a^ mean, median, SD								
Rotavirus	$12.83, 7.4, 23.37	$7.62, 4.15, 17.52	$22.66, 14.59, 26.02	$7.15, 3.34, 13.8	$18.7, 15.14, 12.62	$11.27, 6.84, 10.16	$15.59, 9.5, 38.38	$6.2, 1.71, 23.87
*Cryptosporidium*	$11.81, 5.71, 23.2	$5.62, 3.72, 5.91	$22.69, 12.92, 43.02	$7.22, 3.79, 8.44	$19.75, 16.91, 21.41	$7.6, 4.5, 8.43	$13.36, 7.9, 14.16	$4.09, 1.59, 7.47
ST-producing ETEC	$9.38, 4.56, 17.11	$5.65, 3.72, 6.4	$15.17, 11.08, 17.05	$4.78, 3.45, 4.91	$18.51, 16.33, 13.37	$7.24, 4.53, 7.55	$10.04, 5.56, 12.85	$5.33, 1.41, 28.06
*Shigella*	$10.61, 5.76, 25.64	$7.95, 3.54, 21.77	$17.18, 12.51, 18.06	$4.92, 2.96, 5.26	$15.52, 13.87, 8.71	$8.55, 5.27, 8.51	$11.17, 6.83, 11.51	$8.68, 1.62, 59.63
*Campylobacter jejuni*	$8.59, 4.22, 27.71	$5.6, 3.92, 6.01	$22.49, 13, 38.06	$7.75, 3.91, 7.35	$16.89, 14.19, 20.58	$6.76, 4.55, 6.48	$8.86, 4.99, 10.52	$5.59, 1.54, 39.99
Norovirus GII	$8.19, 4.5, 10.63	$6.11, 3.58, 7.18	$13.29, 9.66, 15.16	$7.42, 3.15, 8.66	$18.89, 15.19, 11.98	$6.31, 4.58, 5.31	$10.84, 5.29, 14.69	$2.89, 1.41, 5
*Vibrio cholerae O1*	$8.67, 4.75, 11.03	$-, -, -	$-, -, -	$9.63, 6.14, 9.04	$22.33, 21.59, 10.43	$10.06, 6.4, 9.32	$10.92, 9.5, 9.25	$6.58, 2.38, 11.73
LSD any pathogen	$5.03, 3.03, 21.4	$3.36, 2.82, 2.85	$7.53, 4.58, 11.35	$3.58, 2.68, 10.96	$-, -, -	$3.76, 3.38, 2.41	$6.54, 4.4, 36.3	$4.87, 1.11, 38.68
MSD any pathogen	$11.84, 7.08, 18.63	$6.83, 4.02, 14.17	$18.02, 11.51, 29.23	$8.55, 4.45, 11.53	$18.31, 15.18, 14.13	$8.06, 5.08, 9.21	$11.98, 7.5, 12.53	$4.89, 1.64, 15.01
Lost work time, d	0.8, 0, 1.6	1.0, 0.5, 1.7	0.1, 0, 0.5	1.6, 0.7, 2.3	2.4, 2, 1.7	0.3, 0, 0.6	1.0, 0.2, 2.2	0.3, 0, 0.9
Percent inpatient	14.4%	19.4%	0.9%	41.9%	10.9%	15.3%	30.7%	0.6%
Length of stay, d	3.6, 3, 2.8	3.1, 3, 1.8	6.2, 3.5, 5.8	4.8, 4, 4.3	2.4, 2, 1.4	2.0, 2, 1.0	4.2, 4, 1.9	2.9, 2, 3.1
Inpatient total costs, mean, median, SD								
Rotavirus	$18.7, 12.82, 21.45	$14.52, 8.02, 29.57	$169.89, 167.36, 53.02	$10.34, 7.34, 10.23	$33.1, 28, 19.34	$19.53, 16.74, 11.59	$19.8, 13.51, 13.66	$7.1, 7.1, 0.97
*Cryptosporidium*	$19.47, 13.57, 23.14	$11.13, 8.33, 9.2	$122.19, 129.12, 64.68	$11.52, 8.5, 10.19	$32.04, 27.29, 16.03	$19.72, 17.05, 12.62	$22.52, 16.31, 17.27	$16.19, 16.2, 1.74
ST-producing ETEC	$17.2, 11.51, 17.38	$12.02, 7.98, 11.67	$48.37, 48.37, 10.39	$9, 5.82, 7.38	$36.37, 29.17, 26.87	$18.44, 16.72, 11.72	$19.03, 13.12, 18.19	$9.6, 9.6, 4.49
*Shigella*	$17.94, 11.89, 14.86	$12.33, 9.47, 9.67	$-, -, -	$11.85, 9.73, 6.83	$26.05, 25.44, 11	$18.49, 14.96, 11.74	$18.93, 11.77, 16.02	$9.73, 9.73, 11.58
*Campylobacter jejuni*	$18.94, 12.3, 27.43	$9.62, 4.78, 10.35	$275.67, 275.67, -	$12.82, 9.56, 8.2	$24.81, 20.86, 12.22	$15.86, 14.26, 9.91	$20.52, 12.58, 20.08	$6.49, 6.28, 6.12
Norovirus GII	$19.89, 13.44, 18.3	$12.73, 9.52, 10.91	$90.48, 90.48, -	$14.91, 11.98, 10.45	$36.94, 34.91, 15.25	$15.2, 16.04, 8.29	$25.28, 14.6, 23.51	$-, -, -
*Vibrio cholerae O1*	$14.89, 11.68, 10.73	$-, -, -	$-, -, -	$10.28, 9.23, 9.18	$26.01, 28.84, 12.48	$15.64, 14.65, 11.12	$14.09, 11.11, 9.89	$13.47, 13.47, 1.37
LSD any pathogen	$0, 0, 0	$0, 0, 0	$0, 0, 0	$0, 0, 0	$0, 0, 0	$0, 0, 0	$0, 0, 0	$0, 0, 0
MSD any pathogen	$19.01, 12.4, 27.31	$11.63, 7.87, 17.49	$151.95, 117.3, 133.02	$11.03, 8.28, 9.98	$31.82, 25.88, 20.44	$18.74, 16.07, 13.25	$19.76, 12.75, 16.29	$15.33, 12.5, 15.13
Outpatient total costs, mean, median, SD								
Rotavirus	$10.52, 5.41, 23.69	$4.39, 3.66, 3.41	$20.88, 14.56, 19.79	$4.44, 2.65, 15.75	$15.92, 14.3, 8.44	$6.29, 5.13, 4.34	$9.82, 4.71, 56.51	$6.2, 1.71, 23.94
*Cryptosporidium*	$10.09, 4.67, 22.87	$3.68, 3.34, 1.84	$20.57, 12.47, 39.97	$3.47, 2.97, 3.6	$17.85, 14.59, 21.54	$4.91, 4.16, 3.44	$6.57, 4.69, 4.66	$3.93, 1.54, 7.38
ST-producing ETEC	$8.21, 4.07, 16.76	$4.22, 3.46, 3.02	$14.78, 11.04, 16.74	$3.01, 2.79, 1.12	$16.35, 15.01, 8.57	$4.98, 4.24, 3.33	$5.21, 4.4, 3.57	$5.3, 1.38, 28.17
*Shigella*	$8.44, 4.69, 27.68	$6.46, 3.13, 24.41	$17.18, 12.51, 18.06	$2.67, 2.59, 1.15	$14.74, 13.26, 8.03	$5.54, 4.63, 3.8	$7.42, 5.14, 5.45	$8.67, 1.62, 59.91
*Campylobacter jejuni*	$7.54, 3.81, 27.54	$4.02, 3.74, 1.44	$17.42, 12.85, 11.99	$3.53, 3.27, 2.3	$16.45, 13.89, 20.88	$4.93, 4.25, 3.3	$6.73, 4.58, 5.4	$5.58, 1.54, 40.15
Norovirus GII	$6.52, 4.03, 7.71	$4.65, 3.44, 5.06	$12.26, 9.58, 12.31	$2.84, 2.68, 0.89	$16.58, 14.48, 9.34	$5.1, 4.35, 3.29	$6.19, 4.7, 4.78	$2.89, 1.41, 5
*Vibrio cholerae O1*	$6.4, 3.13, 10.27	$-, -, -	$-, -, -	$2.48, 2.48, -	$17.41, 19.34, 5.4	$5.75, 4.81, 4.19	$4.57, 4.25, 1.67	$6.46, 2.32, 11.8
LSD any pathogen	$5.03, 3.03, 21.4	$3.36, 2.82, 2.85	$7.53, 4.58, 11.35	$3.58, 2.68, 10.96	$-, -, -	$3.76, 3.38, 2.41	$6.54, 4.4, 36.3	$4.87, 1.11, 38.68
MSD any pathogen	$10.26, 5.86, 15.68	$5.05, 3.65, 12.26	$16.52, 11.35, 21.67	$4.17, 3.06, 12.74	$16.66, 14.32, 12.18	$5.5, 4.59, 5.4	$7.21, 4.91, 5.54	$4.81, 1.64, 14.98
Drug costs, mean, median, SD								
Rotavirus	$2.88, 0.76, 6.29	$0.33, 0, 1	$12.03, 8.52, 11.24	$0.16, 0.18, 0.17	$0.3, 0, 0.84	$2.19, 1.9, 1.63	$2.37, 1.83, 1.74	$0.07, 0, 0.69
*Cryptosporidium*	$2.74, 0.39, 5.94	$0.21, 0, 1	$9.99, 6.98, 9.66	$0.2, 0.19, 0.33	$0.21, 0, 0.77	$1.78, 1.64, 1.16	$2.37, 1.99, 1.94	$0.06, 0, 0.74
ST-producing ETEC	$1.71, 0, 4.11	$0.19, 0, 0.83	$8.29, 6.43, 7.29	$0.24, 0.18, 0.42	$0.21, 0, 0.82	$1.69, 1.62, 1.08	$1.78, 1.59, 0.96	$0.01, 0, 0.23
*Shigella*	$1.7, 1.72, 2.66	$0.34, 0, 1.07	$9.7, 7.18, 7.54	$0.16, 0.18, 0.23	$0.15, 0, 0.45	$2.16, 1.87, 1.94	$2.2, 1.99, 1.65	$0, 0, 0
*Campylobacter jejuni*	$1.27, 0, 4.54	$0.08, 0, 0.36	$11.43, 6.56, 19.61	$0.2, 0.18, 0.36	$0.14, 0, 0.64	$1.81, 1.67, 1.09	$1.96, 1.96, 0.73	$0.03, 0, 0.49
Norovirus GII	$1.37, 0, 3.65	$0.16, 0, 0.72	$7.66, 5.53, 9.42	$0.22, 0.21, 0.37	$0.25, 0, 0.95	$1.73, 1.53, 1.22	$1.72, 1.72, 0.94	$0, 0, 0
*Vibrio cholerae O1*	$0.92, 0, 1.44	$-, -, -	$-, -, -	$0.11, 0.12, 0.11	$1, 0, 1.99	$2.04, 1.83, 1.26	$2.92, 2.71, 1.74	$0.03, 0, 0.23
LSD any pathogen	$1.29, 0.49, 2.56	$0.01, 0, 0.1	$3.94, 2.35, 4.38	$0.22, 0.18, 0.45	$-, -, -	$1.3, 1.21, 0.45	$1.23, 1.59, 0.56	$0.01, 0, 0.25
MSD any pathogen	$2.97, 1.32, 6.56	$0.29, 0, 1.1	$9.56, 6.48, 11	$0.17, 0.21, 0.15	$0.22, 0, 0.95	$1.9, 1.77, 1.23	$2.32, 1.99, 1.98	$0.02, 0, 0.32
Consultation costs, mean, median, SD								
Rotavirus	$0.32, 0, 0.62	$0, 0, 0	$1.21, 1.26, 0.95	$0, 0, 0.05	$0.08, 0, 0.12	$0.02, 0, 0.02	$0.65, 0.82, 0.3	$0.02, 0, 0.3
*Cryptosporidium*	$0.27, 0, 0.6	$0, 0, 0.01	$0.99, 1.15, 0.94	$0, 0, 0	$0.07, 0, 0.11	$0.01, 0, 0.05	$0.56, 0.41, 0.27	$0, 0, 0.04
ST-producing ETEC	$0.17, 0, 0.48	$0.01, 0, 0.14	$0.77, 0, 0.95	$0, 0, 0	$0.08, 0, 0.12	$0.02, 0, 0.03	$0.53, 0.33, 0.3	$0, 0, 0.02
*Shigella*	$0.32, 0.31, 0.37	$0.01, 0, 0.15	$0.8, 0.98, 0.85	$0, 0, 0.01	$0.08, 0, 0.13	$0.02, 0, 0.04	$0.54, 0.41, 0.27	$0, 0, 0.01
*Campylobacter jejuni*	$0.13, 0, 0.31	$0, 0, 0	$0.97, 1.15, 0.89	$0, 0, 0	$0.08, 0, 0.14	$0.01, 0, 0.02	$0.45, 0.38, 0.19	$0, 0, 0.03
Norovirus GII	$0.13, 0, 0.33	$0, 0, 0	$0.61, 0, 0.74	$0, 0, 0	$0.09, 0, 0.13	$0.01, 0, 0.02	$0.48, 0.33, 0.27	$0, 0, 0.02
*Vibrio cholerae O1*	$0.08, 0, 0.23	$-, -, -	$-, -, -	$0, 0, 0	$0, 0, 0	$0.02, 0, 0.02	$0.73, 0.84, 0.27	$0.01, 0, 0.03
LSD any pathogen	$0.12, 0, 0.35	$0, 0, 0	$0.34, 0, 0.64	$0, 0, 0.09	$-, -, -	$0.01, 0, 0.05	$0.31, 0.31, 0.04	$0.01, 0, 0.25
MSD any pathogen	$0.29, 0, 0.59	$0, 0, 0.07	$0.85, 0.98, 0.95	$0, 0, 0.04	$0.07, 0, 0.12	$0.02, 0, 0.04	$0.57, 0.41, 0.27	$0.01, 0, 0.08
Diagnostics costs, mean, median, SD								
Rotavirus	$0.43, 0, 15.16	$0, 0, 0	$0.21, 0, 0.79	$0.69, 0, 11.53	$0.07, 0, 0.14	$0.01, 0, 0.13	$1.83, 0, 36.61	$0, 0, 0
*Cryptosporidium*	$0.12, 0, 1.06	$0.01, 0, 0.13	$0.38, 0, 1.98	$0, 0, 0	$0.06, 0, 0.12	$0.06, 0, 0.99	$0.2, 0, 0.98	$0, 0, 0
ST-producing ETEC	$0.05, 0, 0.62	$0.09, 0, 1.26	$0.16, 0, 0.58	$0, 0, 0	$0.04, 0, 0.12	$0, 0, 0	$0.08, 0, 0.56	$0, 0, 0
*Shigella*	$0.1, 0, 0.73	$0, 0, 0	$0.1, 0, 0.46	$0, 0, 0	$0.05, 0, 0.12	$0, 0, 0	$0.18, 0, 0.99	$0, 0, 0
*Campylobacter jejuni*	$0.04, 0, 0.59	$0, 0, 0	$0.88, 0, 2.85	$0, 0, 0	$0.05, 0, 0.12	$0.01, 0, 0.12	$0, 0, 0	$0, 0, 0
Norovirus GII	$0.05, 0, 0.65	$0.02, 0, 0.28	$0.26, 0, 1.87	$0, 0, 0	$0.04, 0, 0.11	$0, 0, 0	$0.12, 0, 0.76	$0, 0, 0
*Vibrio cholerae O1*	$0.01, 0, 0.13	$-, -, -	$-, -, -	$0, 0, 0	$0, 0, 0	$0.03, 0, 0.25	$0, 0, 0	$0, 0, 0
LSD any pathogen	$0.34, 0, 13.65	$0.03, 0, 0.8	$0.05, 0, 0.32	$0.67, 0, 10.82	$-, -, -	$0, 0, 0	$1.78, 0, 36.17	$0, 0, 0
MSD any pathogen	$0.14, 0, 3.14	$0.2, 0, 6.99	$0.31, 0, 2.91	$0.03, 0, 0.9	$0.08, 0, 0.69	$0.1, 0, 3.32	$0.12, 0, 0.81	$0, 0, 0
Food costs, mean, median, SD								
Rotavirus	$0.45, 0, 1.4	$0.18, 0, 0.8	$0.19, 0, 1.48	$0.13, 0, 0.78	$0.16, 0, 0.39	$0.33, 0, 0.9	$1.9, 1.83, 2.36	$0.01, 0, 0.1
*Cryptosporidium*	$0.26, 0, 1.18	$0.1, 0, 0.59	$0.23, 0, 1.72	$0.07, 0, 0.46	$0.17, 0, 0.69	$0.22, 0, 0.71	$1.54, 0, 2.21	$0.03, 0, 0.25
ST-producing ETEC	$0.15, 0, 0.69	$0.1, 0, 0.64	$0.05, 0, 0.31	$0.04, 0, 0.25	$0.15, 0, 0.34	$0.13, 0, 0.64	$1.07, 0, 1.81	$0.01, 0, 0.05
*Shigella*	$0.7, 0, 6.89	$1.72, 0, 20.34	$0, 0, 0.01	$0.06, 0, 0.32	$0.11, 0, 0.27	$0.16, 0, 0.47	$0.95, 0, 1.62	$0, 0, 0.04
*Campylobacter jejuni*	$0.17, 0, 0.79	$0.18, 0, 0.9	$0.22, 0, 1.34	$0, 0, 0.01	$0.1, 0, 0.28	$0.17, 0, 0.59	$0.56, 0, 1.46	$0.01, 0, 0.09
Norovirus GII	$0.19, 0, 0.75	$0.14, 0, 0.6	$0.09, 0, 0.77	$0, 0, 0	$0.17, 0, 0.53	$0.12, 0, 0.46	$0.75, 0, 1.47	$0.03, 0, 0.19
*Vibrio cholerae O1*	$0.41, 0, 0.99	$-, -, -	$-, -, -	$0.03, 0, 0.1	$1.46, 0.73, 1.73	$0.43, 0, 0.92	$1.91, 2.14, 1.78	$0.11, 0, 0.34
LSD any pathogen	$0.01, 0, 0.38	$0, 0, 0.02	$0.04, 0, 0.72	$0, 0, 0.04	$-, -, -	$0, 0, 0	$0.02, 0, 0.48	$0, 0, 0
MSD any pathogen	$0.33, 0, 3.52	$0.32, 0, 7.01	$0.12, 0, 1.34	$0.39, 0, 7.59	$0.16, 0, 0.47	$0.17, 0, 0.61	$1.18, 0, 1.91	$0.11, 0, 3.42
Transportation costs, mean, median, SD								
Rotavirus	$0.94, 0, 8.67	$0.66, 0, 2	$0.43, 0, 1.7	$1.06, 0, 3.98	$0.23, 0, 0.85	$1.03, 0.47, 1.37	$1.65, 0.79, 2.3	$1.26, 0, 22.86
Cryptosporidium	$0.69, 0, 6.12	$0.57, 0, 1.11	$0.46, 0, 2.06	$1.09, 0, 2.56	$1.4, 0, 17.07	$0.55, 0.36, 0.79	$1.37, 0.49, 2.61	$0.02, 0, 0.09
ST-producing ETEC	$0.74, 0, 12.64	$0.39, 0, 0.92	$0.24, 0, 0.86	$0.56, 0, 1.58	$0.37, 0, 2.56	$0.51, 0.28, 0.81	$1.18, 0.53, 1.43	$1.75, 0, 27.26
Shigella	$1.29, 0.24, 22.31	$0.53, 0, 1.07	$0.24, 0, 0.67	$0.57, 0, 1.45	$0.14, 0, 0.31	$0.68, 0.25, 1	$1.07, 0.45, 1.73	$4.15, 0, 58.84
*Campylobacter jejuni*	$1.33, 0, 25.26	$0.4, 0, 0.64	$0.9, 0, 5.79	$1.48, 0, 2.73	$1.53, 0, 18.22	$0.54, 0.37, 0.79	$0.61, 0.36, 0.83	$2.18, 0, 39.54
Norovirus GII	$0.46, 0, 1.38	$0.63, 0, 2.14	$0.12, 0, 0.59	$0.58, 0, 1.34	$0.23, 0, 0.81	$0.46, 0.3, 0.65	$1.12, 0.43, 1.89	$0.06, 0, 0.34
Vibrio cholerae O1	$0.57, 0, 1.65	$-, -, -	$-, -, -	$2.66, 0, 5.47	$0, 0, 0	$1.04, 0.39, 1.39	$1.04, 0.67, 1.23	$0.05, 0, 0.22
LSD any pathogen	$0.58, 0, 15.65	$0.41, 0, 1.31	$0.32, 0, 7.46	$0.07, 0, 0.2	$-, -, -	$0.41, 0.4, 0.32	$0.59, 0.37, 0.83	$1.73, 0, 38.28
MSD any pathogen	$0.62, 0, 5.45	$0.51, 0, 1.38	$0.3, 0, 1.75	$1.42, 0, 3.8	$0.36, 0, 5.76	$0.77, 0.37, 4.09	$1.08, 0.46, 1.67	$0.47, 0, 12.15
Lost earning costs, mean, median, SD								
Rotavirus	$5.05, 0.69, 10.67	$4.59, 1.4, 16.67	$2.89, 0.07, 10.71	$4.32, 2.06, 6.43	$14.75, 11.6, 11.2	$4.62, 0.13, 7.59	$5.09, 0.59, 10.39	$2.24, 0.11, 5.88
*Cryptosporidium*	$5.01, 0.64, 17.37	$2.69, 1.14, 4.5	$4.81, 0.07, 34.31	$5.06, 2.46, 7.19	$14.93, 13.36, 11.55	$2.7, 0.13, 6.45	$5.53, 0.59, 10.78	$2.04, 0.11, 5.88
ST-producing ETEC	$4.44, 0.69, 9.03	$2.95, 1.12, 5.44	$2.04, 0.03, 10.18	$2.93, 1.28, 4.16	$14.84, 12.79, 11.02	$2.87, 0.13, 6.1	$3.71, 0.59, 10.14	$1.9, 0.11, 6.47
*Shigella*	$4.58, 0.59, 8.47	$3.6, 1.1, 6.46	$2.19, 0.03, 10.3	$3.21, 1.28, 4.7	$12.19, 10.43, 8.34	$3, 0.13, 5.71	$4.65, 0.59, 9.01	$2.17, 0.11, 6.73
*Campylobacter jejuni*	$3.7, 0.22, 7.53	$3.11, 1.4, 5.31	$2.1, 0.07, 4.35	$5.48, 3.05, 5.55	$12.14, 10.43, 7.65	$1.98, 0.13, 4.89	$3.93, 0.59, 9.05	$1.87, 0.11, 6.41
Norovirus GII	$3.95, 0.57, 8.35	$3.38, 1.4, 6.02	$0.92, 0.03, 3.31	$5.63, 1.57, 7.77	$15.39, 11.98, 11.27	$1.62, 0.13, 3.61	$4.82, 0.59, 12.32	$1.41, 0.11, 4.72
*Vibrio cholerae O1*	$4.08, 0.22, 7.74	$-, -, -	$-, -, -	$5.9, 1.84, 7.29	$17.67, 18.3, 8.98	$3.87, 0.25, 7.03	$2.81, 0.15, 7.4	$3.42, 0.11, 7.46
LSD any pathogen	$0.91, 0.13, 3.11	$1.31, 1.07, 2.13	$0.46, 0.03, 2.94	$1.3, 1.23, 1.85	$-, -, -	$0.39, 0.13, 1.83	$0.48, 0.15, 1.1	$1.7, 0.11, 5.89
MSD any pathogen	$4.95, 0.59, 11.58	$3.48, 1.14, 9.3	$2.45, 0.03, 16.33	$5.9, 2.75, 7.21	$14.47, 11.6, 11.59	$2.63, 0.13, 5.71	$5.13, 0.59, 9.89	$2.19, 0.11, 6.24
Costs before study, mean, median, SD								
Rotavirus	$2.7, 1.21, 5.71	$1.6, 0.68, 2.46	$6.3, 1.54, 11.22	$0.22, 0, 0.56	$0.82, 0.34, 1.08	$3.29, 2.53, 2.9	$1.19, 0.98, 1.11	$2.98, 1.09, 5.65
*Cryptosporidium*	$3.36, 0.89, 7.66	$2.84, 2.12, 2.59	$6.95, 1.39, 12.24	$0.06, 0, 0.17	$0.75, 0.34, 1.3	$2.76, 2.53, 2.2	$0.99, 0.61, 0.97	$2.55, 0.91, 4.75
ST-producing ETEC	$1.74, 0.5, 3.49	$1.73, 1.14, 1.96	$3.48, 1.18, 5.47	$0.07, 0, 0.13	$0.71, 0.22, 1.1	$1.97, 1.27, 1.79	$0.7, 0.46, 1.02	$2.14, 0.53, 4.08
*Shigella*	$1.23, 0.52, 4.85	$1.31, 0.88, 1.49	$3.49, 0.72, 6.41	$0.21, 0.15, 0.26	$0.67, 0.35, 0.76	$2.99, 1.5, 3.43	$0.74, 0.49, 0.76	$3.95, 0.56, 14.58
*Campylobacter jejuni*	$1.48, 0.37, 4.18	$0.05, 0.05, 0.03	$7.02, 1.84, 13.46	$0, 0, -	$0.26, 0.06, 0.4	$2.49, 2.03, 2.21	$0.51, 0.24, 0.7	$1.31, 0.41, 2.69
Norovirus GII	$1.37, 0.6, 2.77	$0.99, 0.07, 1.42	$3.79, 0.97, 7.31	$-, -, -	$1.22, 0.35, 2.15	$2.43, 1.52, 2.43	$0.86, 0.55, 0.82	$0.83, 0.34, 1.2
*Vibrio cholerae O1*	$2.83, 0.86, 8.83	$-, -, -	$-, -, -	$0, 0, 0	$0.44, 0.44, -	$2.44, 2.53, 1.33	$0.87, 0.61, 0.86	$3.75, 0.8, 11.36
LSD any pathogen	$1.43, 0.59, 2.64	$1.62, 0.31, 2.64	$2.76, 0.82, 4.67	$0, 0, 0	$-, -, -	$2.27, 1.72, 2.53	$0.9, 0.49, 0.95	$1.43, 0.32, 2.64
MSD any pathogen	$2.46, 0.75, 7.07	$1.87, 0.88, 2.77	$5.09, 1.04, 11.93	$0.13, 0, 0.37	$0.75, 0.33, 1.13	$2.7, 2.17, 2.37	$0.84, 0.5, 0.92	$2.68, 0.75, 7.51
Costs for treatment at home, mean, median, SD								
Rotavirus	$1.74, 1.59, 1.03	$1.63, 1.73, 0.94	$2.8, 2.75, 0.87	$0.83, 0.66, 0.68	$2.76, 2.75, 0.75	$1.98, 2.05, 0.74	$1.06, 1.02, 0.59	$1.12, 0.79, 0.52
*Cryptosporidium*	$1.81, 1.73, 0.99	$1.66, 1.73, 0.82	$2.56, 2.62, 0.95	$0.92, 0.98, 0.75	$2.75, 2.75, 0.7	$1.89, 1.72, 0.61	$0.99, 1.02, 0.56	$1.1, 0.79, 0.55
ST-producing ETEC	$1.73, 1.71, 0.95	$1.75, 1.73, 0.86	$2.52, 2.59, 0.92	$1.04, 1.03, 0.7	$2.53, 2.56, 0.75	$1.91, 1.72, 0.7	$1.12, 1.02, 0.52	$1.04, 0.79, 0.48
*Shigella*	$1.39, 1.02, 0.83	$1.6, 1.73, 0.91	$2.68, 2.62, 0.86	$0.98, 0.93, 0.66	$2.66, 2.61, 0.77	$1.96, 2.12, 0.66	$1.06, 1.02, 0.52	$1.12, 1.02, 0.49
*Campylobacter jejuni*	$1.56, 1.49, 0.85	$1.84, 1.96, 1.02	$2.61, 2.62, 0.84	$0.84, 0.66, 0.71	$2.67, 2.62, 0.81	$1.92, 1.72, 0.63	$1.11, 1.02, 0.4	$1.08, 0.79, 0.48
Norovirus GII	$1.68, 1.59, 0.86	$1.71, 1.73, 0.85	$2.43, 2.43, 0.8	$0.99, 1.03, 0.7	$2.55, 2.56, 0.82	$1.93, 2.05, 0.64	$1.16, 1.02, 0.51	$1.08, 0.79, 0.48
*Vibrio cholerae O1*	$1.42, 1.49, 0.72	$-, -, -	$-, -, -	$0.94, 0.93, 0.73	$2.35, 2.18, 0.44	$1.97, 2.14, 0.66	$0.89, 0.83, 0.66	$1.21, 1.02, 0.52
LSD any pathogen	$1.48, 1.49, 0.69	$1.54, 1.73, 0.48	$1.87, 1.73, 0.91	$1.32, 1.36, 0.57	$-, -, -	$1.56, 1.49, 0.64	$1.35, 1.49, 0.42	$1.05, 0.79, 0.49
MSD any pathogen	$1.85, 1.73, 1	$1.78, 1.77, 0.94	$2.52, 2.56, 0.86	$0.75, 0.66, 0.67	$2.72, 2.62, 0.8	$1.98, 1.86, 0.67	$1.02, 1.02, 0.5	$1.09, 0.79, 0.5
Other costs, mean, median, SD								
Rotavirus	$0.14, 0, 0.81	$0.05, 0, 0.37	$0.36, 0, 1.03	$0, 0, 0	$0.29, 0, 1.62	$0.29, 0, 0.64	$0, 0, 0	$0, 0, 0.01
*Cryptosporidium*	$0.13, 0, 0.69	$0.01, 0, 0.14	$0.35, 0, 1.16	$0, 0, 0	$0.1, 0, 0.27	$0, 0, -	$0, 0, 0	$0, 0, 0
ST-producing ETEC	$0.06, 0, 0.37	$0.01, 0, 0.07	$0.17, 0, 0.73	$0, 0, 0	$0.11, 0, 0.33	$0, 0, 0	$0, 0, 0	$0, 0, 0
*Shigella*	$0.01, 0, 0.15	$0.01, 0, 0.14	$0.1, 0, 0.55	$0, 0, 0	$0.1, 0, 0.27	$0, 0, 0	$0, 0, 0	$0, 0, 0.03
*Campylobacter jejuni*	$0.03, 0, 0.23	$0, 0, 0	$0.22, 0, 0.92	$0, 0, 0	$0.1, 0, 0.13	$-, -, -	$0, 0, 0	$0, 0, 0
Norovirus GII	$0.03, 0, 0.26	$0, 0, 0	$0.07, 0, 0.58	$0, 0, 0	$0.12, 0, 0.38	$-, -, -	$0, 0, 0	$0, 0, 0.01
*Vibrio cholerae O1*	$0.01, 0, 0.05	$-, -, -	$-, -, -	$-, -, -	$0.15, 0.15, 0.16	$0, 0, 0	$0, 0, 0	$0, 0, 0.01
LSD any pathogen	$0, 0, 0.06	$0, 0, 0	$0.01, 0, 0.11	$0, 0, 0	$-, -, -	$0, 0, -	$0, 0, 0	$0, 0, 0
MSD any pathogen	$0.09, 0, 0.55	$0.02, 0, 0.23	$0.19, 0, 0.78	$0, 0, 0	$0.14, 0, 0.72	$0.07, 0, 0.31	$0, 0, 0	$0, 0, 0.02

Abbreviations: ETEC, enterotoxigenic *Escherichia coli*; LSD, less severe diarrhea; MSD, moderate to severe diarrhea; ST, heat-stable toxin.

^a^Component costs may not exactly sum to total costs because 340 patients reported a total cost only, and component costs were not included.

Missing values (-) indicate that no patient with cost data in that country had that pathogen.

## Appendix

**Table:** Appendix Table 3. Results of 2-Part Regression Models by Country

	Coefficient	Standard Error	Z	*P*>|z|	95% Confidence Interval
Total					
Probability of any costs					
Rotavirus	0.46	0.08	5.74	0.00	0.30–0.62
*Cryptosporidium*	0.01	0.09	0.14	0.89	–0.17–0.19
ST-producing ETEC	–0.05	0.09	–0.59	0.56	–0.23–0.13
*Shigella*	0.29	0.10	2.74	0.01	0.08–0.49
*Campylobacter jejuni*	–0.30	0.10	–3.16	0.00	–0.49– –0.11
Norovirus_GII	0.10	0.11	0.89	0.37	–0.11–0.31
*Vibrio cholerae* O1	0.86	0.19	4.43	0.00	0.48–1.24
Gender (female)	–0.05	0.06	–0.84	0.40	–0.16–0.07
Age group (ref: infant)					
Toddler	–0.18	0.07	–2.65	0.01	–0.31– –0.05
Child	–0.37	0.08	–4.67	0.00	–0.53– –0.22
Country					
Mali	5.20	0.20	25.57	0.00	4.80–5.60
Mozambique	2.82	0.12	22.83	0.00	2.58–3.06
Kenya	2.58	0.10	26.98	0.00	2.39–2.76
India	7.08	0.58	12.21	0.00	5.94–8.22
Bangladesh	0.00	(empty)	0.00	0.00	0.00–0.00
Pakistan	–0.23	0.07	–3.31	0.00	–0.36– –0.09
Constant	–0.17	0.07	–2.36	0.02	–0.31– –0.03
Expected costs given any costs					
Rotavirus	0.36	0.04	9.03	0.00	0.28–0.43
*Cryptosporidium*	0.16	0.05	3.41	0.00	0.07–0.26
ST-producing ETEC	0.01	0.06	0.18	0.86	–0.10–0.12
*Shigella*	0.29	0.05	5.25	0.00	0.18–0.39
*Campylobacter jejuni*	0.01	0.06	0.19	0.85	–0.10–0.12
Norovirus_GII	–0.06	0.07	–0.93	0.35	–0.20–0.07
*Vibrio cholerae* O1	0.38	0.13	2.93	0.00	0.13–0.64
Gender (female)	0.04	0.03	1.40	0.16	–0.02–0.10
Age group (ref: infant)					
Toddler	–0.10	0.03	–2.96	0.00	–0.17– –0.03
Child	–0.22	0.04	–5.63	0.00	–0.29– –0.14
Country					
Mali	0.60	0.06	9.52	0.00	0.48–0.73
Mozambique	–0.29	0.07	–3.91	0.00	–0.44– –0.14
Kenya	0.77	0.07	11.03	0.00	0.63–0.90
India	–0.26	0.06	–4.04	0.00	–0.39– –0.13
Bangladesh	0.11	0.07	1.59	0.11	–0.03–0.24
Pakistan	0.04	0.08	0.55	0.58	–0.12–0.20
Constant	2.16	0.06	34.73	0.00	2.04–2.28
Gambia					
Probability of any costs					
Rotavirus	0.41	0.13	3.21	0.00	0.16–0.66
*Cryptosporidium*	0.25	0.14	1.70	0.09	–0.04–0.53
ST-producing ETEC	0.09	0.15	0.59	0.55	–0.20–0.38
*Shigella*	0.49	0.17	2.87	0.00	0.15–0.82
*Campylobacter jejuni*	0.32	0.33	0.96	0.34	–0.33–0.97
Norovirus_GII	0.07	0.16	0.42	0.68	–0.24–0.37
*Vibrio cholerae* O1	0.00	(omitted)	0.00	0.00	0.00–0.00
Gender (female)	–0.06	0.09	–0.69	0.49	–0.25–0.12
Age group (ref: infant)					
Toddler	–0.16	0.11	–1.56	0.12	–0.37–0.04
Child	–0.25	0.13	–1.91	0.06	–0.51–0.01
Constant	–0.25	0.10	–2.58	0.01	–0.44– –0.06
Expected costs given any costs					
Rotavirus	0.33	0.16	2.03	0.04	0.01–0.65
*Cryptosporidium*	0.00	0.19	–0.01	0.99	–0.37–0.37
ST-producing ETEC	0.01	0.20	0.03	0.97	–0.38–0.39
*Shigella*	0.41	0.22	1.90	0.06	–0.01–0.83
*Campylobacter jejuni*	–0.13	0.41	–0.32	0.75	–0.94–0.68
Norovirus_GII	0.08	0.21	0.39	0.70	–0.33–0.49
Gender (female)	0.00	0.12	0.02	0.98	–0.24–0.25
Age group (ref: infant)					
Toddler	0.02	0.14	0.16	0.88	–0.25–0.30
Child	–0.02	0.18	–0.11	0.91	–0.37–0.33
Constant	2.09	0.13	16.64	0.00	1.84–2.34
Mali					
Expected costs					
Rotavirus	0.33	0.08	4.10	0.00	0.17–0.49
*Cryptosporidium*	0.36	0.09	4.07	0.00	0.19–0.53
ST-producing ETEC	–0.01	0.12	–0.04	0.96	–0.24–0.23
*Shigella*	0.27	0.21	1.28	0.20	–0.14–0.69
*Campylobacter jejuni*	0.33	0.21	1.57	0.12	–0.08–0.75
Norovirus_GII	–0.11	0.18	–0.62	0.54	–0.45–0.23
Gender (female)	–0.02	0.05	–0.45	0.65	–0.13–0.08
Age group (ref: infant)					
Toddler	–0.30	0.06	–4.74	0.00	–0.42– –0.18
Child	–0.47	0.07	–7.08	0.00	–0.60– –0.34
Constant	2.91	0.06	52.90	0.00	2.80–3.02
Mozambique					
Expected costs					
Rotavirus	–0.04	0.10	–0.39	0.69	–0.24–0.16
*Cryptosporidium*	0.07	0.12	0.60	0.55	–0.16–0.31
ST-producing ETEC	–0.24	0.15	–1.56	0.12	–0.53–0.06
*Shigella*	–0.12	0.21	–0.55	0.58	–0.54–0.30
*Campylobacter jejuni*	0.05	0.33	0.17	0.87	–0.59–0.70
Norovirus_GII	–0.02	0.28	–0.05	0.96	–0.57–0.54
*Vibrio cholerae* O1	0.92	0.56	1.64	0.10	–0.18–2.02
Gender (female)	0.15	0.09	1.72	0.09	–0.02–0.33
Age group (ref: infant)					
Toddler	–0.43	0.10	–4.17	0.00	–0.63– –0.23
Child	–0.49	0.13	–3.94	0.00	–0.74– –0.25
Constant	2.11	0.09	23.39	0.00	1.94–2.29
Kenya					
Probability of any costs					
Rotavirus	0.23	0.26	0.89	0.37	–0.28–0.74
*Cryptosporidium*	–0.14	0.25	–0.54	0.59	–0.63–0.36
ST-producing ETEC	–0.10	0.27	–0.38	0.70	–0.62–0.42
*Shigella*	0.08	0.33	0.25	0.80	–0.56–0.73
*Campylobacter jejuni*	–0.58	0.24	–2.45	0.01	–1.04– –0.12
Norovirus_GII	0.17	0.41	0.42	0.67	–0.63–0.97
Gender (female)	0.14	0.17	0.83	0.41	–0.19–0.47
Age group (ref: infant)					
Toddler	–0.10	0.20	–0.51	0.61	–0.48–0.29
Child	–0.04	0.21	–0.18	0.86	–0.45–0.37
Constant	2.31	0.16	14.35	0.00	2.00–2.63
Expected costs given any costs					
Rotavirus	–0.03	0.06	–0.49	0.62	–0.14–0.08
*Cryptosporidium*	0.06	0.06	0.91	0.36	–0.07–0.18
ST-producing ETEC	–0.01	0.06	–0.18	0.85	–0.14–0.11
*Shigella*	–0.15	0.07	–2.09	0.04	–0.30– –0.01
*Campylobacter jejuni*	–0.09	0.07	–1.40	0.16	–0.22–0.04
Norovirus_GII	–0.02	0.09	–0.25	0.80	–0.19–0.15
*Vibrio cholerae* O1	0.18	0.29	0.61	0.54	–0.39–0.74
Gender (female)	0.01	0.04	0.25	0.80	–0.07–0.09
Age group (ref: infant)					
Toddler	–0.01	0.05	–0.27	0.79	–0.10–0.08
Child	–0.16	0.05	–3.39	0.00	–0.26– –0.07
Constant	3.00	0.04	81.32	0.00	2.93–3.07
India					
Expected costs					
Rotavirus	0.63	0.06	10.88	0.00	0.52–0.74
*Cryptosporidium*	0.10	0.07	1.47	0.14	–0.03–0.24
ST-producing ETEC	0.10	0.10	1.01	0.31	–0.09–0.29
*Shigella*	0.33	0.11	3.03	0.00	0.12–0.55
*Campylobacter jejuni*	–0.08	0.07	–1.15	0.25	–0.22–0.06
Norovirus_GII	–0.02	0.09	–0.27	0.79	–0.21–0.16
*Vibrio cholerae* O1	0.57	0.15	3.78	0.00	0.27–0.86
Gender (female)	–0.09	0.04	–1.94	0.05	–0.17–0.00
Age group (ref: infant)					
Toddler	0.04	0.05	0.79	0.43	–0.06–0.14
Child	–0.06	0.06	–1.06	0.29	–0.18–0.05
Constant	1.81	0.05	40.04	0.00	1.72–1.90
Bangladesh					
Expected costs					
Rotavirus	0.52	0.09	5.93	0.00	0.35–0.69
*Cryptosporidium*	0.16	0.13	1.22	0.22	–0.10–0.43
ST-producing ETEC	–0.02	0.16	–0.13	0.90	–0.34–0.30
*Shigella*	0.15	0.08	1.92	0.06	0.00–0.31
*Campylobacter jejuni*	–0.15	0.10	–1.40	0.16	–0.35–0.06
Norovirus_GII	0.07	0.14	0.50	0.62	–0.21–0.35
*Vibrio cholerae* O1	0.11	0.32	0.34	0.73	–0.52–0.75
Gender (female)	0.06	0.06	0.99	0.32	–0.06–0.19
Age group (ref: infant)					
Toddler	0.04	0.08	0.54	0.59	–0.11–0.20
Child	0.08	0.09	0.84	0.40	–0.10–0.26
Constant	2.16	0.06	33.25	0.00	2.03–2.28
Bangladesh					
Probability of any costs					
Rotavirus	0.65	0.12	5.30	0.00	0.41–0.90
*Cryptosporidium*	–0.16	0.15	–1.10	0.27	–0.46–0.13
ST-producing ETEC	–0.22	0.15	–1.51	0.13	–0.51–0.07
*Shigella*	0.25	0.15	1.65	0.10	–0.05–0.55
*Campylobacter jejuni*	–0.29	0.11	–2.62	0.01	–0.51– –0.07
Norovirus_GII	0.06	0.17	0.37	0.71	–0.27–0.39
*Vibrio cholerae* O1	1.08	0.20	5.37	0.00	0.69–1.47
Gender (female)	–0.12	0.09	–1.34	0.18	–0.30–0.06
Age group (ref: infant)					
Toddler	–0.24	0.11	–2.32	0.02	–0.45– –0.04
Child	–0.58	0.13	–4.34	0.00	–0.84– –0.32
Constant	–0.31	0.09	–3.35	0.00	–0.49– –0.13
Pakistan					
Expected costs given any costs					
Rotavirus	0.06	0.18	0.34	0.74	–0.29–0.42
*Cryptosporidium*	–0.23	0.24	–0.97	0.33	–0.71–0.24
ST-producing ETEC	0.29	0.24	1.18	0.24	–0.19–0.76
*Shigella*	0.75	0.25	2.96	0.00	0.25–1.24
*Campylobacter jejuni*	0.18	0.19	0.96	0.34	–0.19–0.54
Norovirus_GII	–0.59	0.26	–2.24	0.03	–1.10– –0.07
*Vibrio cholerae* O1	0.22	0.27	0.82	0.41	–0.31–0.75
Gender (female)	0.38	0.15	2.56	0.01	0.09–0.67
Age group (ref: infant)					
Toddler	–0.14	0.17	–0.84	0.40	–0.48–0.19
Child	–0.11	0.22	–0.51	0.61	–0.55–0.32
Constant	2.04	0.14	14.39	0.00	1.77–2.32

Abbreviations: ETEC, enterotoxigenic *Escherichia coli*; LSD, less severe diarrhea; MSD, moderate to severe diarrhea; ST, heat-stable toxin.

## Appendix

**Table:** Appendix Table 4. Results of 2-Part Regression Models by Inpatient/Outpatient and MSD/LSD

	Coefficient	Standard Error	Z	*P*>|z|	95% Confidence Interval
MSD inpatient					
Probability of any costs					
Rotavirus	0.33	0.23	1.42	0.15	–0.12–0.78
*Cryptosporidium*	0.42	0.28	1.50	0.13	–0.13–0.96
ST-producing ETEC	0.16	0.33	0.49	0.63	–0.49–0.81
*Shigella*	0.42	0.35	1.20	0.23	–0.26–1.10
*Campylobacter jejuni*	–0.13	0.54	–0.25	0.81	–1.19–0.92
Norovirus_GII	0.42	0.38	1.10	0.27	–0.33–1.17
*Vibrio cholerae* O1	–1.71	0.60	–2.85	0.00	–2.89– –0.54
Gender (female)	–0.27	0.19	–1.44	0.15	–0.64–0.10
Age group (ref: infant)					
Toddler	–0.17	0.21	–0.81	0.42	–0.59–0.25
Child	–0.66	0.27	–2.41	0.02	–1.19– –0.12
Country					
Mali	0.00	(empty)	0.00	0.00	0.00–0.00
Mozambique	2.67	0.26	10.42	0.00	2.16–3.17
Kenya	2.73	0.37	7.36	0.00	2.00–3.45
India	0.00	(empty)	0.00	0.00	0.00–0.00
Bangladesh	0.00	(empty)	0.00	0.00	0.00–0.00
Pakistan	1.69	0.89	1.90	0.06	–0.05–3.44
Constant	0.57	0.21	2.68	0.01	0.15–0.99
Expected costs given any costs					
Rotavirus	0.01	0.05	0.16	0.87	–0.08–0.10
*Cryptosporidium*	0.03	0.06	0.47	0.64	–0.09–0.14
ST-producing ETEC	–0.04	0.08	–0.54	0.59	–0.20–0.11
*Shigella*	–0.03	0.06	–0.44	0.66	–0.15–0.10
*Campylobacter jejuni*	–0.11	0.09	–1.32	0.19	–0.28–0.05
Norovirus_GII	0.12	0.09	1.28	0.20	–0.06–0.31
*Vibrio cholerae* O1	–0.11	0.13	–0.87	0.38	–0.36–0.14
Gender (female)	0.07	0.04	1.78	0.07	–0.01–0.15
Age group (ref: infant)					
Toddler	–0.11	0.05	–2.40	0.02	–0.20– –0.02
Child	–0.09	0.06	–1.55	0.12	–0.21–0.02
Country					
Mali	2.24	0.17	13.09	0.00	1.90–2.57
Mozambique	–0.34	0.07	–4.76	0.00	–0.48– –0.20
Kenya	0.72	0.09	8.28	0.00	0.55–0.89
India	0.19	0.07	2.64	0.01	0.05–0.34
Bangladesh	0.25	0.07	3.61	0.00	0.11–0.39
Pakistan	0.14	0.27	0.53	0.60	–0.39–0.67
Constant	2.78	0.07	39.80	0.00	2.64–2.92
MSD outpatient					
Probability of any costs					
Rotavirus	0.35	0.10	3.47	0.00	0.15–0.54
*Cryptosporidium*	–0.06	0.11	–0.49	0.63	–0.28–0.17
ST-producing ETEC	–0.10	0.12	–0.84	0.40	–0.33–0.13
*Shigella*	0.29	0.12	2.38	0.02	0.05–0.52
*Campylobacter jejuni*	–0.35	0.11	–3.26	0.00	–0.56– –0.14
Norovirus_GII	0.06	0.13	0.48	0.64	–0.19–0.32
*Vibrio cholerae* O1	0.80	0.21	3.88	0.00	0.40–1.21
Gender (female)	0.02	0.07	0.21	0.83	–0.13–0.16
Age group (ref: infant)					
Toddler	–0.12	0.08	–1.42	0.16	–0.28–0.04
Child	–0.17	0.10	–1.71	0.09	–0.36–0.02
Country					
Mali	8.22	1.00	8.20	0.00	6.26–10.19
Mozambique	2.50	0.21	11.70	0.00	2.08–2.91
Kenya	2.55	0.11	23.57	0.00	2.34–2.76
India	7.07	0.71	9.95	0.00	5.68–8.47
Bangladesh	0.00	(empty)	0.00	0.00	0.00–0.00
Pakistan	–0.03	0.09	–0.33	0.74	–0.20–0.14
Constant	–0.27	0.09	–2.89	0.00	–0.45– –0.09
Expected costs given any costs					
Rotavirus	0.13	0.04	2.89	0.00	0.04–0.22
*Cryptosporidium*	0.07	0.05	1.46	0.14	–0.02–0.17
ST-producing ETEC	0.03	0.06	0.51	0.61	–0.09–0.15
*Shigella*	0.12	0.06	2.10	0.04	0.01–0.23
*Campylobacter jejuni*	–0.04	0.05	–0.81	0.42	–0.15–0.06
Norovirus_GII	–0.10	0.07	–1.42	0.16	–0.24–0.04
*Vibrio cholerae* O1	0.09	0.14	0.65	0.52	–0.19–0.37
Gender (female)	0.05	0.03	1.74	0.08	–0.01–0.11
Age group (ref: infant)					
Toddler	–0.10	0.04	–2.70	0.01	–0.17– –0.03
Child	–0.18	0.04	–4.50	0.00	–0.26– –0.10
Country					
Mali	0.78	0.07	10.85	0.00	0.64–0.92
Mozambique	–0.60	0.11	–5.56	0.00	–0.82– –0.39
Kenya	0.82	0.08	10.87	0.00	0.68–0.97
India	–0.31	0.07	–4.22	0.00	–0.46– –0.17
Bangladesh	–0.07	0.08	–0.83	0.41	–0.23–0.09
Pakistan	0.16	0.09	1.81	0.07	–0.01–0.33
Constant	2.05	0.07	28.27	0.00	1.91–2.19
LSD					
Probability of any costs					
Rotavirus	0.60	0.18	3.28	0.00	0.24–0.95
*Cryptosporidium*	–0.27	0.20	–1.36	0.17	–0.67–0.12
ST-producing ETEC	0.05	0.18	0.30	0.76	–0.30–0.41
*Shigella*	–0.78	0.35	–2.20	0.03	–1.47– –0.08
*Campylobacter jejuni*	–0.29	0.25	–1.15	0.25	–0.79–0.21
Norovirus_GII	0.08	0.24	0.34	0.73	–0.38–0.54
*Vibrio cholerae* O1	1.71	0.79	2.17	0.03	0.17–3.25
Gender (female)	0.02	0.12	0.15	0.88	–0.22–0.26
Age group (ref: infant)					
Toddler	–0.36	0.14	–2.53	0.01	–0.64– –0.08
Child	–0.66	0.17	–3.94	0.00	–0.98– –0.33
Country					
Mali	4.06	0.23	17.80	0.00	3.61–4.50
Mozambique	3.21	0.21	15.52	0.00	2.81–3.62
India	7.15	1.01	7.10	0.00	5.18–9.12
Bangladesh	0.00	(empty)	0.00	0.00	0.00–0.00
Pakistan	–0.36	0.15	–2.45	0.01	–0.65– –0.07
Constant	0.00	0.00	0.00	0.00	0.00–0.00
Expected costs given any costs					
Rotavirus	0.54	0.13	4.22	0.00	0.29–0.80
*Cryptosporidium*	0.10	0.17	0.62	0.54	–0.22–0.43
ST-producing ETEC	0.00	0.16	0.02	0.99	–0.32–0.32
*Shigella*	0.80	0.32	2.50	0.01	0.17–1.43
*Campylobacter jejuni*	0.32	0.27	1.16	0.25	–0.22–0.85
Norovirus_GII	0.01	0.19	0.06	0.95	–0.36–0.38
*Vibrio cholerae* O1	0.46	0.72	0.63	0.53	–0.96–1.87
Gender (female)					
Age group (ref: infant)	0.00	0.00	0.00	0.00	0.00–0.00
Toddler	0.02	0.11	0.22	0.83	–0.18–0.23
Child					
Country	0.00	0.00	0.00	0.00	0.00–0.00
Mali	0.45	0.18	2.55	0.01	0.10–0.80
Mozambique	–0.38	0.19	–1.99	0.05	–0.75– –0.01
India	–0.23	0.18	–1.25	0.21	–0.58–0.13
Bangladesh	0.04	0.19	0.18	0.85	–0.34–0.41
Pakistan	0.63	0.26	2.38	0.02	0.11–1.14
Constant	1.49	0.17	8.69	0.00	1.15–1.82

Abbreviations: ETEC, enterotoxigenic *Escherichia coli*; LSD, less severe diarrhea; MSD, moderate to severe diarrhea; ST, heat-stable toxin.
